# Imaging brain change across different time scales

**DOI:** 10.3389/fninf.2012.00029

**Published:** 2012-12-21

**Authors:** Jussi Tohka, Ulla Ruotsalainen

**Affiliations:** Department of Signal Processing, Tampere University of TechnologyTampere, Finland

Brain imaging can be used to characterize changes occurring in a brain during very different time-scales. Anatomical brain imaging can be used to probe the changes in brains between different species due to evolution (e.g., Schoenemann et al., 2005) and, at the other end of the spectrum, functional imaging with electroencephalography (EEG) and magnetoencephalography (MEG) can be used to study very rapid changes in brain states due to sensory processing, cognition, and social interaction (Hari et al., 2010). Although the time-scales, imaging techniques, and analysis methodologies are different, the fundamental methodological challenge is the same: how to quantitatively measure the change in brain images acquired. Obviously, the detailed methodological apparatus required to analyze the data depends on the question of interest and the nature of the data. In a very general level, one can aim to characterize the brain change within an individual over a certain period of time, perhaps in a response to external stimuli, or between two or more groups (cross-sectional studies) or even ask whether the change trajectories differ between two or more groups. With this research topic entitled “Imaging brain change across different time scales,” we hope to bring together several different reports on the theme to provide a good possibility to observe differences and similarities in analysis strategies required by different research questions and imaging techniques.

This research topic features seven papers, one of which (Henson et al., 2011 edited by Luis M. Martinez) was adopted, at a request from the corresponding author, from the Frontiers in Human Neuroscience to this research topic. In their article, Henson et al. (2011) review methods based on a parametric empirical Bayesian framework to EEG/MEG source reconstruction and illustrate the benefits of multi-modal [EEG, MEG, and functional magnetic resonance imaging (fMRI)] and multi-subject integration when studying cortical responses to faces. Brain changes studied in this work are rapid, occurring in under a second.

Two of the papers focus on functional MRI. Kiviniemi et al. (2009) study the hyperventilation induced alterations on fractal metrics, such as 1/f trend constant, fractal dimension, and Hurst exponent, derived from the blood oxygenation level dependent (BOLD) signal with resting state fMRI. The metrics were found to show statistically significant differences between scans taken before and immediately after hyperventilation. Kauppi et al. (2010) present methods to study differences in intersubject correlation (ISC) in different frequency components of the BOLD signal during movie watching. They found that the frontal and temporal cortical areas show high levels of ISC predominantly at low frequency components of BOLD signals whereas visual cortical areas exhibit ISC also at higher BOLD signal frequencies. Although the research questions addressed by these papers are distinct, there are many similarities in methodological “toolboxes” required by the methods such as frequency decompositions (and wavelet transforms related to this) and computationally intensive non-parametric testing for statistical inference. In these works, the brain change is occurring in the range of seconds to minutes.

Four of the papers focus on the anatomical magnetic resonance imaging (MRI) and address the brain change occurring during a life span of a subject or an animal, in practice the time scale is from days to years. Mietchen and Gaser (2009) represent a review of computational morphometry based strategies including voxel, deformation, and surface based morphometry to detect a brain change; Ziegler et al. (2012) provide an overview of the methods and models to quantify age-related changes in humans based on MRI; Lerch et al. (2012) consider mouse MRI and address the questions about relative benefits of *in-vivo* and *ex-vivo* imaging; finally, MacKenzie-Graham (2012) provides a commentary on the article by Lerch et al. Interestingly, both Lerch and Ziegler consider the benefits and limitations of the cross-sectional vs. longitudinal study designs from different perspectives; the benefit of the better image resolution provided by *ex-vivo* imaging of mice does not benefit human studies based on cross-sectional design and thus the advantages of the longitudinal designs are more imminent in the studies of human aging/development than in similar studies of mice. It is also interesting to note that a high degree of synergy between life span modeling of structural brain change and functional imaging studies exist as the basic machinery in both comes from the time series modeling as is clear, for instance, based on (Henson et al., 2011) and (Ziegler et al., 2012). However, the number of time points is usually much more limited in the case longitudinal anatomical data that makes the assumed parametric form of the “age trajectory” an important choice (Ziegler et al., 2012).

As expected, it is not very difficult to come up with an important research question clearly falling under rather broad title of this research topic but not covered by this research topic. This research topic does not include, for instance, a specific article on challenges when comparing brain imaging data between different species to gain insights on brain change due to evolution although these challenges are touched upon in the review article by Mietchen and Gaser (2009).
